# Optimized MT-Capture targeted enrichment sequencing for the complete genome of norovirus in stool and sewage samples

**DOI:** 10.3389/fmicb.2026.1772465

**Published:** 2026-07-27

**Authors:** Ziyang Hao, Lele He, Kangchen Zhao, Liping Yu, Yuhan Ding, Heng Rong, Shuo Ning, Qiao Qiao, Xiaojuan Zhu, Tao Wu, Jinlei Guo, Ge Hu, Jincan Ke, Yuhua Qi, Jie Ding, Hongbin Zhang, Yiyue Ge, Lunbiao Cui

**Affiliations:** 1 School of Public Health, Nanjing Medical University, Nanjing, China; 2 NHC Key Laboratory of Enteric Pathogenic Microbiology, Jiangsu Provincial Medical Key Laboratory of Pathogenic Microbiology in Emerging Major Infectious Diseases, Jiangsu Provincial Center for Disease Control and Prevention, Nanjing, China; 3 Nanjing Municipal Center for Disease Control and Prevention, Nanjing, China; 4 Jiangyin Municipal Center for Disease Control and Prevention, Wuxi, China

**Keywords:** multiplex PCR, norovirus, optimized MT-Capture, sequencing, targeted enrichment

## Abstract

Human norovirus is a major acute gastroenteritis pathogen with high genotypic diversity, mutation, and recombination rates. Existing sequencing methods for monitoring its genetic variations are limited by low sensitivity and contamination risk. This study optimized our previously established Micro Target Hybrid Capture System (MT-Capture) by increasing nucleic acid input, streamlining library preparation, and introducing a deployable USER enzyme-based contamination prevention system. The optimized protocol completes library preparation and capture within 7 hours. Compared with multiplex PCR sequencing, optimized MT-Capture avoids primer updates, captures multiple genotypes with improved compatibility, and detects low-abundance samples more efficiently. The USER enzyme degrades contaminating amplicons, ensuring data authenticity. The MT-Capture-USER system provides a rapid, sensitive, and contamination-resistant approach for norovirus whole-genome sequencing and epidemiological surveillance.

## 1 Introduction

Human norovirus (HuNoV) is the leading cause of epidemic and sporadic acute gastroenteritis (AGE). It causes around 700 million infections and 200,000 deaths globally each year (Carlson et al., 2024), and accounts for nearly 90% of AGE outbreaks in China (Cao et al., 2022). With no licensed specific treatments or vaccines, HuNoV represents a major public health burden (Carlson et al., 2024).

HuNoV is a non-enveloped, single-stranded positive-sense RNA virus with a 7.5 kb genome containing three open reading frames (ORFs) (van Beek et al., 2018). ORF1 encodes non-structural proteins such as RNA-dependent RNA polymerase (RdRp), while ORF2 and ORF3 encode the major capsid protein VP1 and minor capsid protein VP2, respectively (van Beek et al., 2018). Frequent recombination at the ORF1-ORF2 junction contributes to its high genetic diversity (Chhabra et al., 2019). HuNoV is primarily transmitted via the fecal-oral route with an extremely low infectious dose. Sewage serves as an important transmission and reservoir medium for NoV (Eftim et al., 2017). The genotype distribution in sewage closely reflects human AGE epidemics, supporting the importance of sewage surveillance (Fumian et al., 2019; Yue et al., 2024). However, complex sewage matrices, PCR inhibitors (Mao et al., 2020), low viral loads, mixed genotypes (Fumian et al., 2019; Cao et al., 2022; Yue et al., 2024), and high genetic diversity of NoV collectively hinder viral enrichment, detection, and epidemiological tracing.

Currently, conventional NoV detection and genotyping relies on real-time reverse transcription polymerase chain reaction (RT-qPCR), and reverse transcription polymerase chain reaction (RT-PCR) combined with Sanger sequencing (Marpaung et al., 2025; Srivastava and Prasad, 2023). Limited by specific primers and short amplified fragments, these techniques fail to timely identify mutations or emerging variants, delaying outbreak responses. Thus, establishing a method for continuous monitoring of NoV genomic characteristics in complex samples (stool or sewage) is crucial for AGE prevention, control and vaccine development.

Whole-genome sequencing enables the acquisition of full viral genomes, facilitating NoV genotyping, evolutionary analysis and variant prediction (Oude Munnink et al., 2020; Silva et al., 2021; Tohma et al., 2021). While metagenomic sequencing (mNGS) enables unbiased pathogen detection, its analytical sensitivity is severely compromised by excessive host and microbial background in stool and sewage samples, limiting its utility in large-scale targeted enteric pathogens surveillance. By contrast, targeted enrichment sequencing (tNGS) features faster workflows, higher sensitivity and cost-effectiveness for specific pathogen analysis (Child et al., 2023). Current tNGS strategies mainly include multiplex PCR sequencing and probe capture sequencing. Multiplex PCR sequencing features simple workflow, rapid runtime, and high flexibility, but suffers from amplification bias, primer dimer interference, and reduced sensitivity to new variants due to delayed primer updates (Fitzpatrick et al., 2023; Kazama et al., 2017; Qin et al., 2023; Daviña-Núñez et al., 2024). In contrast, probe capture sequencing offers advantages such as high probe fault tolerance, low amplification bias, and superior sequence consistency (Brown et al., 2016; Strubbia et al., 2019). However, traditional liquid hybridization capture remains cumbersome, time-consuming, and contamination-prone (Schultzhaus et al., 2021), limiting its high-throughput application in clinical and sewage samples.

To address the limitations of traditional probe capture technology, we previously developed the Micro Target Hybrid Capture System (MT-Capture), which simplifies hybridization and elution with non-isometric conjugated probes and achieves a comparable runtime to multiplex PCR. This system has been successfully applied to coronavirus whole-genome sequencing and respiratory pathogen detection (Meng et al., 2024; Qiao et al., 2025; Ma et al., 2026). However, aerosol contamination in routine operations frequently induces false positives, compromising the accuracy and reliability of MT-Capture for HuNoV detection in complex stool and sewage samples. The USER enzyme is a highly effective decontamination tool that specifically degrades dU-labeled contaminant DNA via UDG base excision and APE1 strand cleavage, without affecting target template amplification (Kwok and Higuchi, 1989; Pruvost et al., 2005). Thus, we optimized the MT-Capture system by designing a HuNoV-specific probe panel, optimizing library preparation, and integrating USER enzyme system to establish an efficient and contamination-resistant HuNoV whole-genome sequencing method. Validated with 69 HuNoV-positive stool samples and 20 sewage samples alongside multiplex PCR sequencing as a control, this optimized method efficiently retrieves high-quality full-length HuNoV genomes from low-abundance samples and covers all mutation sites and variants. It provides reliable support for viral evolution analysis, adaptive mutation identification and epidemiological tracing, while lowering contamination risk and improving genome capture stability in complex samples.

## 2 Materials and methods

### 2.1 Sample source

69 HuNoV-positive stool samples and 20 HuNoV-positive sewage samples (covering Genogroups I (GI) and II (GII)) were collected from 2018 to 2024 from multiple sentinel hospitals across different cities and regions in Jiangsu Province, under the Jiangsu Provincial Intestinal Infectious Disease Surveillance Program, Jiangsu Provincial Center for Disease Control and Prevention (Jiangsu Provincial CDC). All procedures involving human-derived samples were approved by the Jiangsu Provincial CDC Ethics Committee.

### 2.2 Virus enrichment, extraction and quantification

Nucleic acid extraction and enrichment were conducted in a Biosafety Level II (BSL-2) laboratory. HuNoV enrichment from sewage samples was performed with the CP-Select Rapid Microbial Enrichment System (INNOVAPREP, United States): 5 mL of environmental water was added to a 0.2 μm hollow-fiber disposable enrichment column (INNOVAPREP, United States, #CC08022), using the HOLLOW program (flow rate: 2 mL/min) and automatic elution (final volume: 200 μL). Clinical stool samples were thawed on ice and vortexed thoroughly for 1 min to ensure homogeneous mixing, then centrifuged at 8000 rpm for 5 min to remove insoluble impurities; and the supernatant was carefully transferred to a new sterile centrifuge tube. Nucleic acid was extracted from 200 μL of each sample via the GeneRotex 96 automatic extractor (Xi’an Tianlong Biotechnology, China, #T335) per the manufacturer’s protocol. HuNoV detection for all samples was performed with the One Step Prime-Script™ RT-PCR Kit (Perfect Real Time; TAKARA, Japan, #RR064A), using primers and probes described previously (Loisy et al., 2005). RT-qPCR reaction system and procedure are provided in Supplementary Table S1. Following experiments used RT-qPCR cycle threshold (Ct) values as viral load indicators (Hadzega et al., 2023), where Ct ≤ 30 denotes medium-to-high viral load, Ct ≤ 35 low viral load, and Ct > 35 extremely low viral load. All samples and extracted RNA were stored at −80 °C.

### 2.3 Optimization of library preparation for targeted enrichment

**Figure 1:**
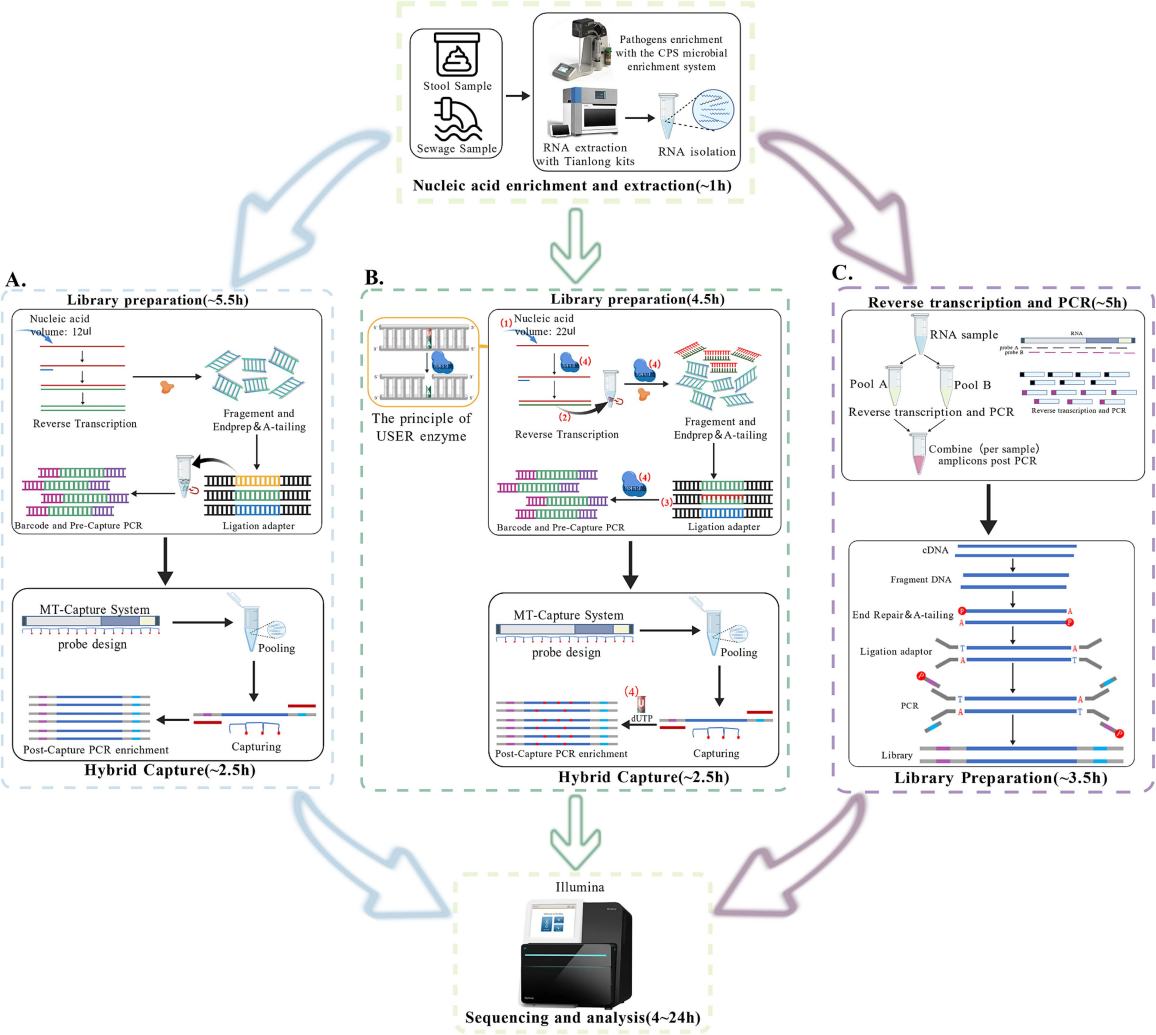
Workflow comparison: pre-optimized MT-Capture, optimized MT-Capture, and multiplex PCR sequencing. (A) Pre-optimized MT-Capture Sequencing: Whole-genome sequencing workflow & specific duration. (B) Optimized MT-Capture sequencing: Whole-genome sequencing workflow, specific duration, and key optimizations: (1) adjusting the sample nucleic acid loading volume; (2) omitting the second-strand synthesis step, directly purifying reverse transcription products after first-strand cDNA synthesis, and then performing fragmentation, end repair & A-tailing after purification; (3) omitting post-adapter ligation purification, and then performing Pre-Capture PCR directly using the adapter ligation products as templates; (4) anti-contamination protocol: USER enzyme and dUTP addition positions. (C) Multiplex PCR sequencing: Whole-genome sequencing workflow & specific duration. Created with BioGDP.com (JJiang et al., 2025).

Library preparation referenced previous studies (Meng et al., 2024) with the following optimizations to improve sensitivity, efficiency, and contamination control: (1) To enhance detection sensitivity for low viral load samples, sample nucleic acid input was adjusted to 22 μL. (2) To streamline workflow and shorten library preparation duration, traditional second-strand synthesis was omitted; first-strand cDNA synthesis products were purified directly, followed by fragmentation, end repair, and A-tailing. (3) Adapter ligation products were used as templates directly for Pre-Capture PCR without additional purification. (4) To reduce library contamination risk, 2.5 μL of 10 mM dUTP was additionally added to the Post-Capture PCR system of hybrid capture libraries to generate U-containing amplicons in the initial experiment. Additionally, USER enzyme (NEB #M5508S) was separately added to three steps in all next experiments, namely RNA denaturation & random primer binding step, end repair & A-tailing step, and Pre-Capture PCR step, to digests residual uracil-containing amplicons from aerosols or previous experiments. Extra digestion steps were, respectively, added before RNA denaturation & random primer binding and Pre-Capture PCR, with the conditions being 25 °C for 5 min and 37 °C for 10 min, respectively (Figure 1B).

### 2.4 Micro target hybrid capture

#### 2.4.1 Probe design

Representative HuNoV genomes, including global variants and dominant Chinese strains, were retrieved from NCBI (Supplementary Table S2) for MT-Capture probe design. Following (Meng et al., 2024), we designed 3,517 unique tiled probes (20–100 nt) without overlap, with additional probes targeting hypervariable regions for complete coverage (Supplementary Table S3). Probe specificity was verified by alignment against NCBI core_nt and human RefSeqGene databases.

#### 2.4.2 Hybrid and enrichment

HuNoV libraries were hybridized with the nov panel using the μCaler® Hybrid Capture Reagent v2 (Nanodigmbio (Nanjing) Biotechnology Co., Ltd., China, #1105201) at 98 °C for 2 min (denaturation) and 60 °C for 1 h (hybridization). Target enrichment was conducted with Streptavidin Beads (Nanodigmbio). Remaining procedures and precautions followed (Meng et al., 2024). To verify the anti-contamination effect of USER enzyme, hybrid capture was performed with probes for human genomic DNA and λDNA.

### 2.5 Library preparation for multiplex PCR sequencing

The commercial Norovirus Whole Genome Amplification Kit (X Company, China) was used. It is divided into GI and GII genogroup-specific types, each targeting the respective whole genome. Reverse transcription-multiplex PCR was performed on extracted nucleic acid per the kit’s protocol (Supplementary Table S4). Subsequently, PCR products were subjected to library preparation with the ILMN DNA LP(M) Barcoded Fragmentation Kit (Illumina, United States, #20060059), taking nearly 8.5 h (Figure 1C).

### 2.6 Sequencing

Samples were sequenced on the Illumina MiniSeq sequencing platform using paired-end 2 × 150 bp sequencing with the Illumina MiniSeq High Output Reagent Cartridge (300 cycles) (Illumina, #15073286) and Illumina MiniSeq Flow Cell (Illumina, #15073184).

### 2.7 Bioinformatics analysis

Raw sequencing data were analyzed using Nanodigmbio’s XCapViz platform with an in-house workflow validated against multiple pathogens (Ma et al., 2026; Meng et al., 2024; Qiao et al., 2025). Briefly, paired-end reads were merged, adapter-trimmed, and quality-filtered using fastp (v0.21.0) (Chen et al., 2018); duplicates were removed using in-house scripts^1^. Reads were aligned to hg19 using BWA (v0.7.17) (Li, 2013) to remove host-derived sequences, and remaining reads were mapped to a custom norovirus reference (GenBank). The top five reference sequences by reads count were selected for assembly using Iterative Refinement Meta-Assembler (IRMA, v1.0.3) (Shepard et al., 2016). For consensus calling, positions with depth ≥20× and Q30 quality were retained; others were masked as “*N*”. Depth and coverage were calculated using samtools (v1.22.1). For mixed GI/GII sewage samples, reads were aligned separately to genotype-specific references; chimeric assemblies were assessed by inspecting genogroup-specific markers, with no artifacts detected. For multiplex PCR data, 30 nucleotides were trimmed from both ends using fastp. Visualization was performed with GraphPad Prism (v8.0.2), with all analyses under default parameters. Genotypes were confirmed using the online norovirus typing tool^2^. To validate assembly accuracy, consensus sequences from our pipeline were compared with those from CLC Genomics Workbench (v22.0, Qiagen); both approaches yielded fully concordant results.

### 2.8 Statistical analysis

Statistical analyses were performed using IBM SPSS Statistics 23. Normality was assessed by the Shapiro–Wilk test. Comparisons between optimized MT-Capture and multiplex PCR used paired *t*-tests for normally distributed data or the paired Wilcoxon signed-rank test for non-normally distributed data. Descriptive statistics are presented as mean ± SD for normal variables and median (IQR) for non-normal variables. Effect sizes were calculated as Cohen’s *d* (*t*-tests) or *r* = *Z*/√*n* (Wilcoxon tests). A two-tailed *p* < 0.05 was considered statistically significant.

## 3 Results

### 3.1 Omitting second-strand synthesis and purification processes shortens the library preparation duration

Although our previously established MT-Capture sequencing technology greatly shortened the time compared to traditional probe hybrid capture, its workflow is still less rapid than multiplex PCR sequencing. To further optimize the workflow for complex practical samples, we attempted to shorten library preparation duration. Results showed omitting two steps had no impact on sequencing efficiency: (1) library preparation second-strand synthesis (replaced by direct purification of reverse transcription products after first-strand cDNA synthesis, followed by fragmentation, end repair & A-tailing); (2) purification after adapter ligation (adapter ligation products used directly as templates for Pre-Capture PCR). Moreover, despite adding USER enzyme digestion steps, library preparation duration was shortened by nearly 1.5 h compared with multiplex PCR library preparation (Figure 1).

### 3.2 Increasing the nucleic acid amount improves sequencing depth and on-target rates

**Figure 2:**
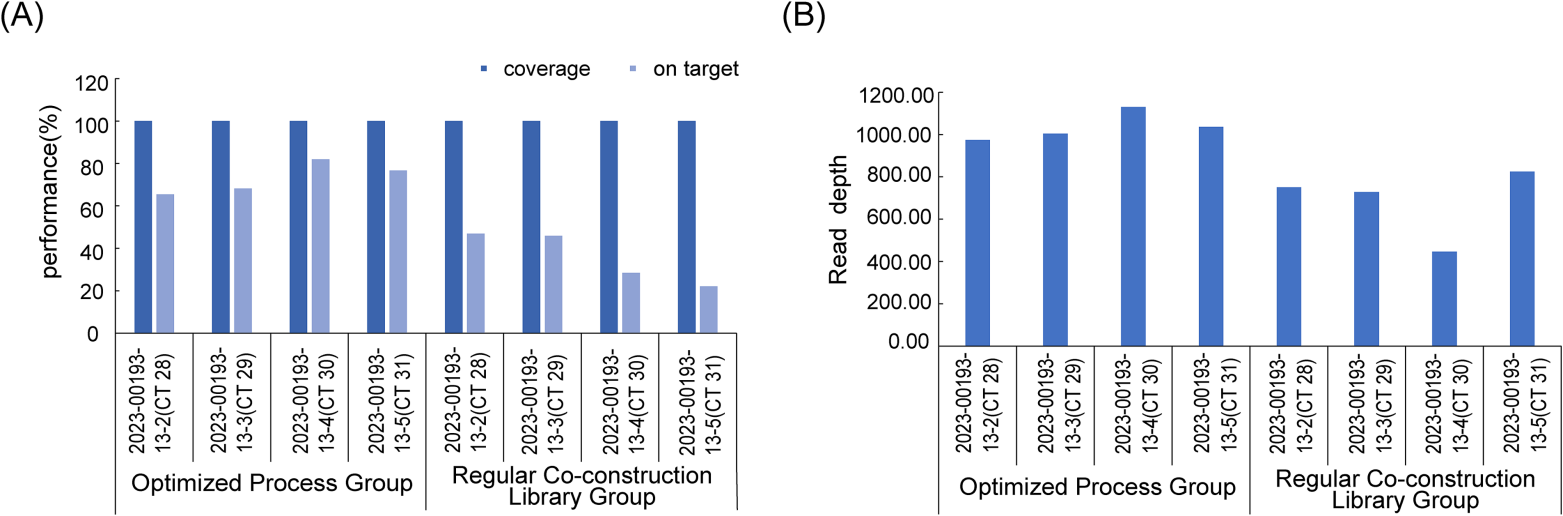
Comparison: pre-optimized vs. optimized MT-capture groups. **(A)** On-target rate and whole-genome coverage. **(B)** Sequencing depth.

To address sequencing challenges caused by high background interference and low viral load, we increased RNA input for first-strand cDNA synthesis from 12 μL to 22 μL, with parallel sequencing of serial dilutions of the same HuNoV-positive stool samples. Results showed both 22 μL and 12 μL RNA inputs achieved 100% whole-genome coverage, but 22 μL RNA significantly improved on-target rates and sequencing depth (Figure 2). Optimized RNA input enhanced detection efficiency for low-viral-load samples.

### 3.3 USER enzyme can effectively prevent the occurrence of contamination

**Figure 3:**
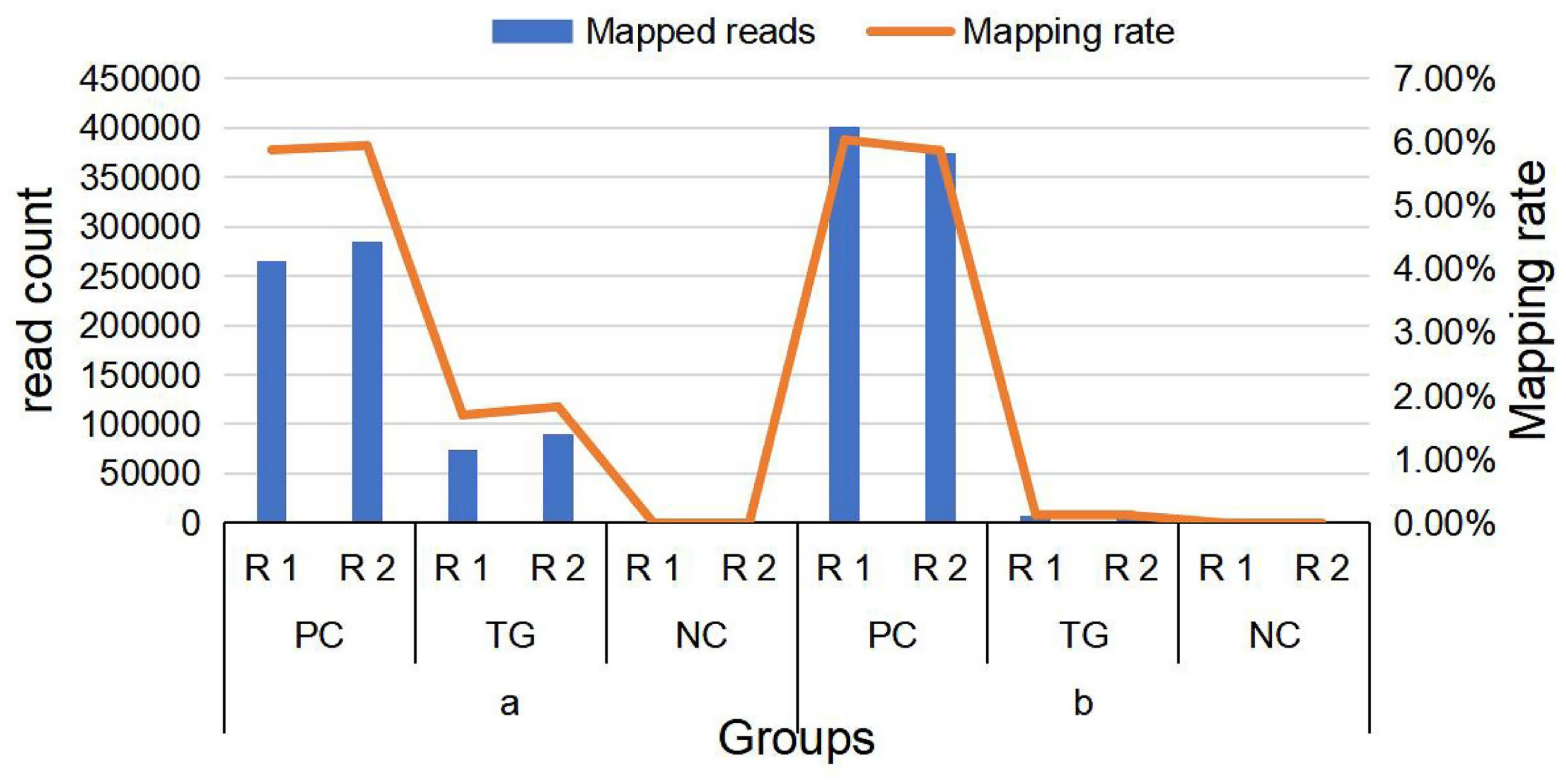
λDNA-aligned data in human gDNA libraries. Libraries were constructed with fragmented human gDNA, and hybrid capture was performed with human and λDNA probes. (a) 1 ng dUTP-containing λDNA contaminant added in end repair & A-tailing; (b) 1 ng dUTP-containing λDNA contaminant added in Pre-Capture PCR; PC, positive control: contaminant model added, without USER enzyme added; TG, test group: both contaminant model and USER enzyme added; NC, negative control: no contaminant model or USER enzyme added.

To evaluate the anti-contamination effect of the USER enzyme, a contamination model was constructed by mixing fragmented human gDNA and λDNA at a 1:1 copy ratio, with hybrid capture performed using human and λDNA probes and dUTP added during post-capture PCR. Three parallel groups were established using pure human gDNA as the starting template: positive control (PC, with contamination model, no USER enzyme), test group (TG, with both contamination model and USER enzyme), and negative control (NC, without either). 1 ng of the contamination model was spiked separately into end repair & A-tailing and Pre-Capture PCR steps, where USER enzyme was also introduced. After library preparation, hybrid capture was performed with human and λDNA probes. Analysis of Index-split data from human gDNA libraries (Figure 3) showed that λDNA was abundant in PC but significantly reduced in TG, decreasing from 5.92 to 1.77% when spiked during end repair & A-tailing (Figure 3A), and from 6.03 to 0.13% when spiked during Pre-Capture PCR (Figure 3B). These results demonstrate that USER enzyme addition before Pre-Capture PCR effectively digests U-containing exogenous contaminants.

### 3.4 Optimized MT-Capture sequencing can capture the whole genomes of multiple genotypes of noroviruses GI and GII

**Figure 4:**
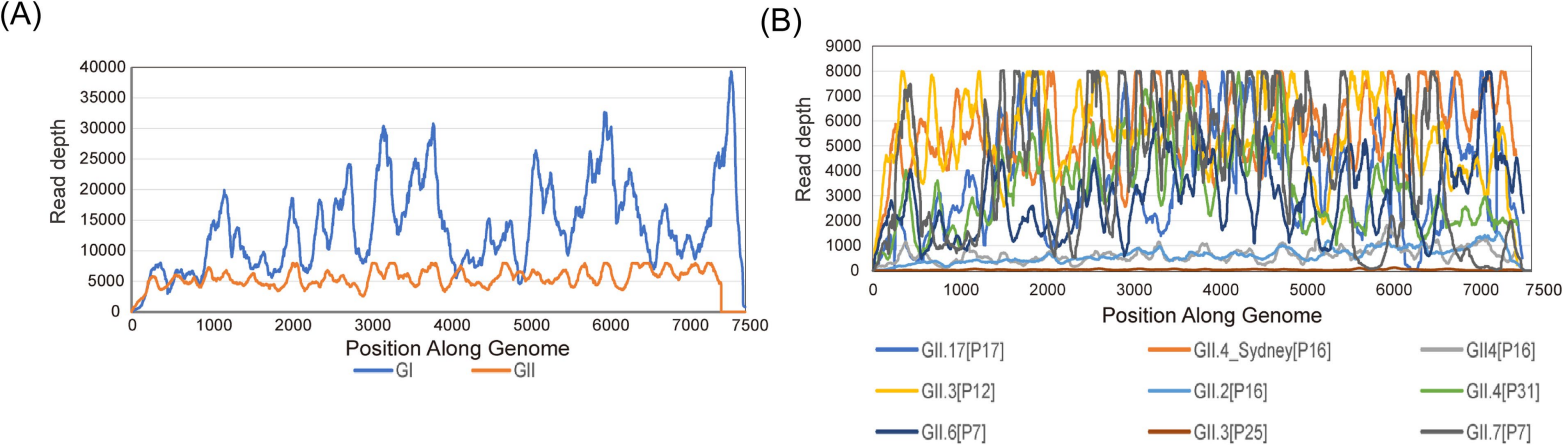
Optimized MT-capture: norovirus GI/GII multiple-genotype whole-genome capture. **(A)** GI/GII: Read depth & whole-genome coverage; **(B)** GII genotypes: Read depth & whole-genome coverage.

To evaluate optimized MT-Capture for norovirus capture, major genotypes from prevalent genogroups (GI, GII) in stool samples were analyzed, including the predominant genotypes GI.3 [P13] (Ct 23) and GII.4_Sydney [P16] (Ct 25) for genogroup comparison. Both GI and GII achieved whole-genome coverage, with distinct sequencing depth distributions across genogroups or genotypes (Figure 4). GI had higher sequencing depth; GII had slightly lower overall depth due to higher Ct values but maintained full genome coverage (Figure 4A). Within GII, all genotypes exhibited uniform and continuous coverage; GII.4[P16] (Ct 27) and GII.2[P16] (Ct 29) had marginally reduced depth compared with other genotypes due to higher Ct values but also achieved full genome coverage (Figure 4B). These results indicate that optimized MT-Capture efficiently captures whole genomes of GI/GII genogroups and multiple GII genotypes, with good compatibility and flexibility for monitoring genotype-specific mutation patterns.

### 3.5 Optimized MT-Capture sequencing exhibits higher sensitivity than multiplex PCR sequencing

**Figure 5:**
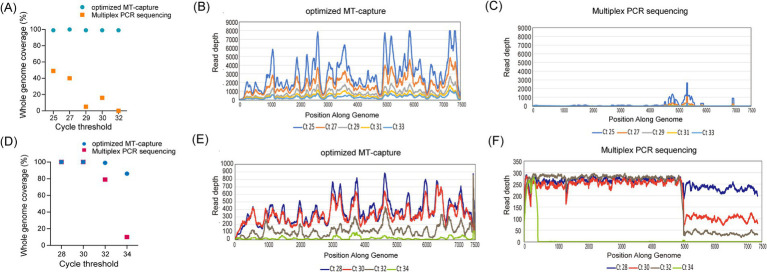
Capture efficiency: Optimized MT-Capture vs. multiplex PCR sequencing. **(A)** GI.3 [P13] whole-genome coverage across CT values (RT-PCR CT = viral load indicator, serially diluted samples, *n* = 1). **(B)** GI.3 [P13] sequencing coverage and depth analyses (optimized MT-Capture, different CT values). **(C)** GI.3 [P13] sequencing coverage and depth analyses (multiplex PCR sequencing, different CT values). **(D)** GII.4_Sydney [P16] whole-genome coverage across CT values (RT-PCR CT = viral load indicator, serially diluted samples, *n* = 1). **(E)** GII.4_Sydney [P16] sequencing coverage and depth analyses (optimized MT-Capture, different CT values). **(F)** GII.4_Sydney [P16] sequencing coverage and depth analyses (multiplex PCR sequencing, different CT values).

To compare norovirus capture efficiency between multiplex PCR sequencing and optimized MT-Capture, single representative strains of GI.3 [P13] and GII.4_Sydney [P16] were independently serially diluted and sequenced via both methods. Results showed optimized MT-Capture maintained ~100% whole-genome coverage across the tested Ct range, with coverage remaining >80% even at Ct 34. In contrast, multiplex PCR coverage decreased with increasing Ct values, with a notable drop at Ct 32 (79.0% vs. 99.85% for optimized MT-Capture) and further decline at Ct 34 (10.35% vs. 86.53%) (Figures 5A,D). Optimized MT-Capture also yielded uniform sequencing depth across the whole genome at different Ct values, sufficient for comprehensive whole-genome analysis, whereas multiplex PCR achieved high depth only in localized genomic regions, particularly for the GI.3 [P13] genotype (Figures 5B,C,E,F). Based on these observations, Ct ≈ 32 was identified as a potential critical threshold for method divergence, which was subsequently validated using independent stool and sewage samples.

**Figure 6:**
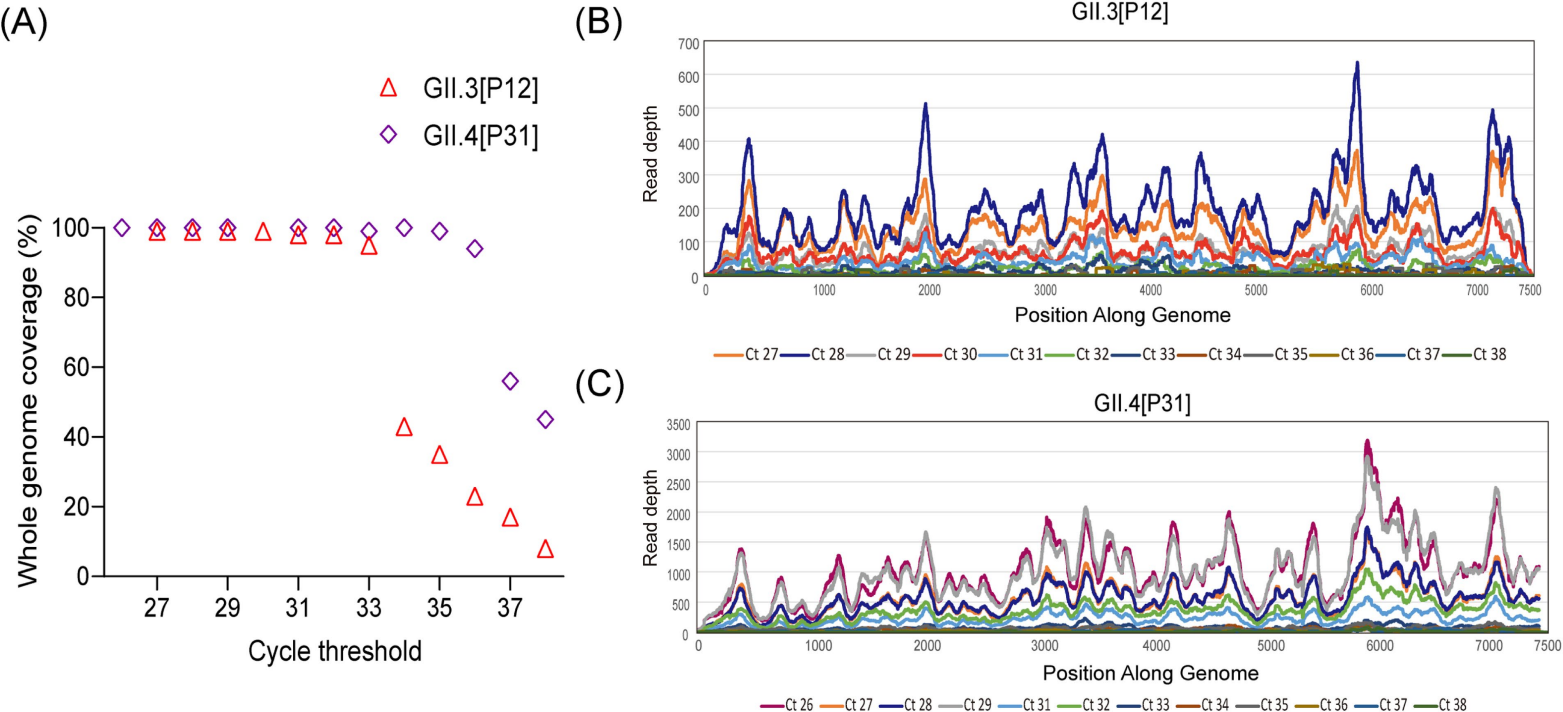
Detection sensitivity of optimized MT-Capture sequencing. **(A)** GII.3 [P12] & GII.4 [P31] whole-genome coverage (CT gradient) (RT-PCR CT = viral load indicator, serially diluted samples). **(B)** GII.3 [P12] sequencing coverage and depth analyses (*n* = 1, across different Ct values). **(C)** GII.4 [P31] sequencing coverage and depth analyses (*n* = 1, across different Ct values).

Furthermore, to further assess the detection sensitivity of optimized MT-Capture, stool samples of common genotypes GII.3 [P12] and GII.4 [P31] were serially diluted and sequenced. The method showed high sensitivity for both genotypes (Figure 6A): GII.3 [P12] maintained 96% whole-genome coverage even at Ct 33, while GII.4 [P31] retained >97% coverage at Ct 37 (extremely low viral load). Uniform sequencing depth was also observed across all tested samples of both genotypes (Figures 6B,C).

### 3.6 Optimized MT-Capture sequencing exhibits stronger stability

**Figure 7:**
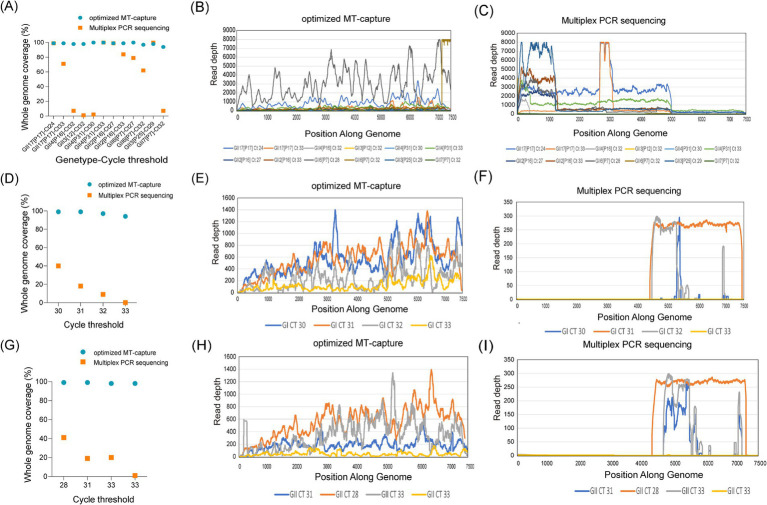
Stability comparison: Optimized MT-Capture vs. multiplex PCR sequencing (low-abundance samples). **(A)** Whole-genome coverage (12 stool samples: distinct genotypes, CT ≈ 30). **(B)** Coverage and sequencing depths|optimized MT-Capture sequencing (12 stool samples, distinct genotypes). **(C)** Coverage and sequencing depths|multiplex PCR sequencing (12 stool samples, distinct genotypes). **(D)** Whole-genome coverage (4 sewage samples: GI genogroup, CT ≈ 30) **(E)** Coverage and sequencing depths|optimized MT-Capture sequencing (4 sewage samples, GI genogroup). **(F)** Coverage and sequencing depths|multiplex PCR sequencing (4 sewage samples, GI genogroup). **(G)** Whole-genome coverage (4 sewage samples, GII genogroup, CT ≈ 30). **(H)** Coverage and sequencing depths|optimized MT-Capture sequencing (4 sewage samples, GII genogroup). **(I)** Coverage and sequencing depths|multiplex PCR sequencing (4 sewage samples, GII genogroup).

To further validate this observed Ct threshold and assess stability, 12 independent stool samples (distinct genotypes, Ct ≈ 30) and 4 independent sewage samples (mixed GI/GII) were sequenced in a paired design. For stool samples, optimized MT-Capture yielded significantly higher coverage than multiplex PCR (Figure 7A; median [IQR]: 99.56% [98.60–99.96%] vs. 75.72% [7.88–99.96%]; Wilcoxon test, *Z* = −2.589, *p* = 0.014, *r* = −0.747). Although sequencing depth did not differ significantly (median [IQR]: 152.19 [78.25–391.29] vs. 613.33 [28.51–971.73]; *p* = 0.424), the higher coverage with MT-Capture reflects superior uniformity, and all 12 stool samples showed smoother coverage profiles (Figures 7B,C). For sewage samples, optimized MT-Capture also achieved significantly higher coverage (mean ± SD: 98.23 ± 1.33% vs. 19.09 ± 14.64%; paired *t*-test, *t* = 10.81, df = 3, *p* = 0.0017, Cohen’s *d* = 5.4) and depth (328.76 ± 172.59 vs. 39.24 ± 46.28; paired *t*-test, *t* = 4.255, df = 3, *p* = 0.024, Cohen’s *d* = 2.128) than multiplex PCR, with consistent results across both genogroups (Figures 7D–I). These results demonstrate that optimized MT-Capture exhibits stronger stability for low-abundance and complex samples.

### 3.7 Optimized MT-Capture sequencing has superior accuracy to multiplex PCR sequencing

**Figure 8:**
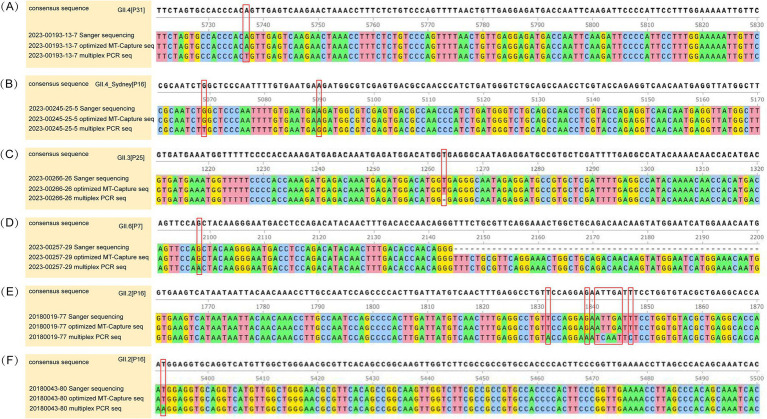
Partial sequencing results of six stool samples representing five different genotypes. **(A)** GII.4[P31]; **(B)** GII.4_Sydney[P16]; **(C)** GII.3[P25]; **(D)** GII.6[P7]; **(E–F)** GII.2[P16]. For each panel, sequences from top to bottom: Sanger sequencing, optimized MT-Capture sequencing, and multiplex PCR sequencing. Red boxes indicate the positions of nucleotide discrepancies.

To compare sequencing accuracy, 7 samples with ≥60% coverage, ≥50 × depth and diverse genotypes were selected from 14 parallel-sequenced specimens for Sanger validation. One GII.17 [P17] sample was excluded due to ambiguous double peaks in Sanger chromatograms, leaving 6 genotypically distinct samples. Optimized MT-Capture matched Sanger sequencing across all validated fragments, significantly outperforming multiplex PCR (Figure 8, Supplementary Table S5). Supplementary Table S6 lists discordant loci, base error rates and regional base concordance rates for each sample, which are limited to partial validated regions due to the 800 bp maximum length of Sanger amplicons. Multiplex PCR exhibited frequent base calling discrepancies, e.g., at position 5,737, the Ct 33 GII.4 [P31] sample called T by multiplex PCR versus A by both MT-Capture and Sanger (Figure 8A); similar discrepancies were observed in GII.4_Sydney [P16], GII.3 [P25], GII.6 [P7] and GII.2 [P16] samples (representative loci in Figures 8B–F). Notably, base differences also existed in genomic regions without Sanger verification, predominantly within the first 300 bp of the 5′-UTR (Supplementary Table S5).

### 3.8 Utilization of optimized MT-Capture sequencing for environmental and clinical samples analysis

**Figure 9:**
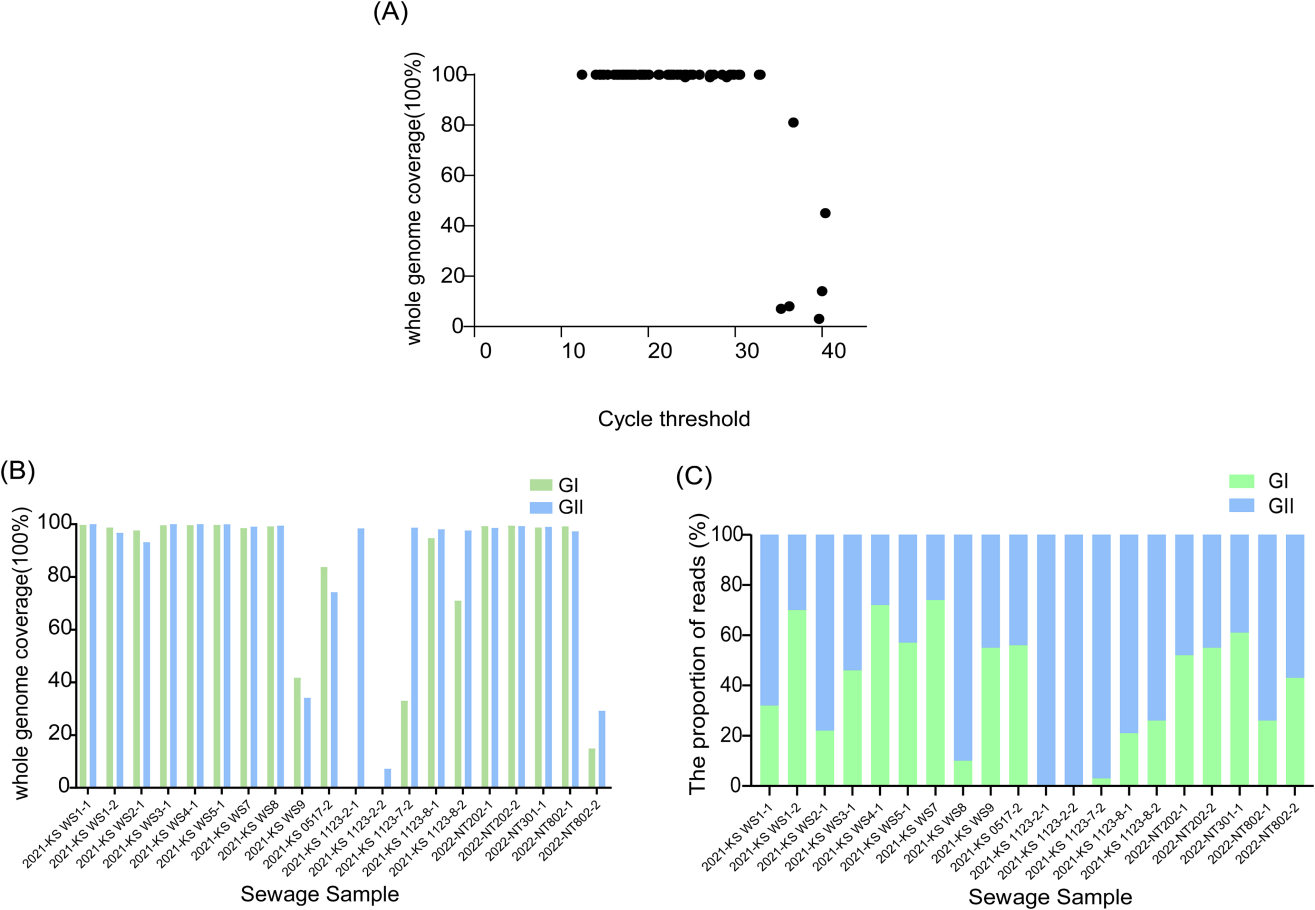
Application results of Optimized MT-Capture for 89 environmental & clinical samples. **(A)** 69 stool samples: Whole-genome coverage. **(B)** 20 sewage samples: GI/GII whole-genome coverage. **(C)** 20 sewage samples: GI/GII reads proportion.

This study applied optimized MT-Capture sequencing to 89 samples (69 stool, 20 sewage; Supplementary Table S7). Results showed stool samples contained 3 genogroups (GI, GII, GIX) and 10 genotypes (1 each for GI/GIX). For GI and GII, all samples with Ct ≤ 33 achieved whole-genome coverage; a complete GIX.1 sequence was also obtained. Reliable viral detection was retained even at extremely low viral load (Ct 36 and Ct 40; Figure 9A). In sewage samples, it enabled simultaneous detection of two norovirus genogroups per sample/reaction (Figure 9B). GII reads predominated in most samples; samples 2021-KS 1123-2-1 and 2021-KS 1123-7-2 yielded almost entirely GII sequences. GI exhibited lower overall whole-genome coverage than GII, but relatively high read proportions in some samples (e.g., 2021-KS WS4-1) (Figure 9C).

## 4 Discussion

HuNoV is a genetically diverse RNA virus that evolves continuously via point mutations and recombination. Multiple genotypes circulate globally, with GII.4 generating new variants every 2–4 years and driving frequent outbreaks (Cao et al., 2022). Therefore, continuous genomic surveillance is critical for NoV prevention, control, and vaccine development. Whole-genome targeted sequencing provides cost-effective genomic data, supporting pathogen typing, key gene analysis, outbreak source tracing, and epidemiological risk prediction (Brown et al., 2016; Child et al., 2023; Silva et al., 2021).

Although MT-Capture outperforms traditional liquid-phase hybridization capture in efficiency and convenience (Meng et al., 2024), its workflow remained longer than multiplex PCR, prompting us to streamline library preparation. Reverse transcription was retained as an essential step for RNA viruses, while second-strand synthesis was omitted after preliminary testing; fragmentation, end-repair, and A-tailing were preserved to maintain recovery from low-abundance samples (Agarwal et al., 2015; Naphade et al., 2022). By introducing first-strand cDNA purification and removing post-adapter ligation purification, we reduced redundant steps without compromising sequencing performance, as validated using clinical stool and sewage samples. The streamlined workflow reduced the total library-to-capture time to 7 h, approximately 1.5 h faster than multiplex PCR under identical conditions. A detailed comparison of the performance characteristics of the three methods is presented in Supplementary Table S8.

Building on this efficiency foundation, we further optimized performance to address key challenges in clinical and environmental samples, particularly low viral loads with high background interference. As increasing nucleic acid input improves sensitivity for low viral loads samples (Moriarty et al., 2021), we raised the input from 12 μL to 22 μL, matching that used in multiplex PCR sequencing. This modification preserved stable whole-genome capture efficiency across all Ct values, while markedly enhancing on-target rates and sequencing depth for low-abundance samples, enabling reliable detection of low viral load specimens.

Beyond efficiency and performance, we optimized MT-Capture to address cross-contamination, a critical challenge in molecular detection. Sequencing contamination is typically linked to inadequate workspace partitioning non-standardized operations (Brown et al., 2016), and aerosol formation from post-amplification tube opening. While multiple anti-contamination strategies exist (Fierer et al., 2025; Morono et al., 2018), the USER enzyme, Uracil-Specific Excision Reagent, offers precise targeting by degrading dU-containing DNA fragments via UDG and APE1 activities without damaging dU-free target templates (Kwok and Higuchi, 1989; Pruvost et al., 2005). We incorporated dUTP during post-capture PCR to label amplicons, and introduced USER enzyme in reverse transcription and library preparation steps to eliminate residual dU-containing carryover. Targeted tests demonstrated that the optimized workflow effectively reduced λDNA contamination in all simulated groups. Given its universal biochemical mechanism, this approach minimizes aerosol-derived carryover in routine sequencing. This is particularly important for HuNoV detection, where even a single contaminating copy can cause false positives, especially in low-titer sewage samples (Chen et al., 2024). In formal sequencing runs, no norovirus-specific reads were detected in negative controls, further confirming that the optimized workflow prevents cross-contamination and ensures data credibility.

The optimized MT-Capture sequencing overcomes critical limitations of conventional methods and performs robustly in practical surveillance. Multiplex PCR, though widely used for viral genome sequencing, is restricted to samples with Ct ≤ 30 and requires separate kits for GI and GII genogroups, preventing mixed-genogroup detection in a single reaction and increasing nucleic acid input, workflow time, and cost. In contrast, optimized MT-Capture system simultaneously captures whole genomes of both GI and GII genogroups and multiple GII genotypes (e.g., GII.4_Sydney [P16], GII.3 [P12], GII.7 [P7]), providing data support for subsequent genotype identification and variation analysis. Minor depth variations may occur across genotypes due to differences in nucleic acid load or extraction efficiency, but these do not compromise overall capture efficiency. Importantly, its optimized sensitivity outperforms multiplex PCR under low-viral-load and high-background conditions.

Raw sewage is a valuable resource for monitoring the genetic diversity of enteric viruses in human populations and plays a critical role in viral disease surveillance (Mao et al., 2020). In this study, multiplex PCR sequencing showed distinct genome coverage patterns between stool and sewage samples: the 5′ end was well covered in stool samples but decreased drastically in sewage samples, whereas the conserved 3′ end remained relatively stable. This may be because viral genomes in stool are relatively intact, supporting efficient amplification of the 5′ region. In sewage, however, viral genomes are degraded under environmental stress, with the highly variable 5′ end being more vulnerable than the conserved 3′ end (Tang et al., 2024). Since multiplex PCR relies on high sequence complementarity between primers and templates, sequence variations and genome damage further compromise amplification efficiency at the 5′ end. Moreover, mixed-genotype infections are common in sewage (Eftim et al., 2017; Fumian et al., 2019; Cao et al., 2022; Yue et al., 2024), further reducing amplification efficiency at the 5′ end. By contrast, our optimized MT-Capture method provided consistent and uniform whole-genome coverage in both sample types, even when viral genomes show partial degradation. It also allowed simultaneous detection of multiple norovirus genogroups within a single sewage sample and generates raw sequencing reads for downstream bioinformatic profiling, supporting preliminary epidemiological investigation of mixed-genogroup infections and routine sewage surveillance.

Multiplex PCR sequencing frequently causes loss of regions near the 5′ or 3′ genomic ends (Tang et al., 2024; Meng et al., 2024), corroborated by our comparative analysis of GII.2 [P16], GII.6 [P7] and GII.4 [P31] samples in which whole-genome discrepancies between the two methods were concentrated in the first 300 bp, highlighting this terminal loss. It also tends to introduce base mismatches during amplification or fragment loss during assembly. In contrast, optimized MT-Capture sequencing is significantly more accurate for genome sequencing than multiplex PCR, attributed to target probes with higher fault tolerance that reduce sequence bias, which was validated by Sanger sequencing, the gold standard for nucleotide sequencing. Notably, MT-Capture still showed base loss in the first 300 bp of GII.3 [P25] samples, likely due to incomplete probe coverage of this genotype’s terminal sequences. Future studies should supplement genotype-specific probes to further improve the technical system.

This study has several limitations. First, our cohort contained limited GI isolates as GII strains predominate locally. Diverse full-length GI reference genomes were incorporated into probe design to capture GI genetic variation. As shown in the Results section, sequencing depth data confirmed superior enrichment efficiency for GI strains. Larger cohorts with balanced GI/GII strains are needed to validate the broad applicability of this workflow. Second, the λDNA model fails to simulate key traits of norovirus aerosol contamination and USER enzyme exclusively removes U-labeled foreign DNA rather than unmodified viral contaminants. Rigorous lab partitioning and standard operations remain essential to avoid contamination. Third, our in-house bioinformatic pipeline, though tested on multiple pathogens, has not been benchmarked against dedicated norovirus analysis workflows. Owing to short-read sequencing, we can recover full-length genomes only from single-genotype stool samples; for mixed sewage samples, resolution is limited to genogroup-level classification, precluding fine-scale genotyping and strain abundance estimation. Additionally, this study centers on targeted viral genome capture and its validation; recombination analysis is not addressed here. Despite these constraints, the high-coverage genomic data are suitable for future evolutionary and recombination surveillance, with further workflow optimization and benchmarking left to subsequent studies.

## 5 Conclusion

In conclusion, the optimized MT-Capture sequencing enables efficient enrichment and genetic variation analysis of HuNoV with high and stable reliability. Integrated with USER enzyme decontamination strategy, this workflow effectively eliminates exogenous amplicon contamination and guarantees the authenticity of sequencing data. Benefiting from its high sensitivity, this method provides a robust technical solution for routine HuNoV surveillance as well as epidemic prevention and control. Future work will introduce external clinical cohorts from different regions to further validate the general applicability and practical value of this approach.

## Data Availability

The data presented in the study are deposited in the National Microbiology Data Center (NMDC) repository, accession number NMDC10020834 (https://nmdc.cn/resource/genomics/project/detail/NMDC10020834).

## Footnotes

1
https://github.com/11ysj11/dedup_fq/tree/main

2
http://www.rivm.nl/mpf/norovirus/typingtool
