# Supplementary material for: Optimized MT-Capture targeted enrichment sequencing for the complete genome of norovirus in stool and sewage samples
Source: Front Microbiol. 2026 Jul 27;17:1772465. doi: 10.3389/fmicb.2026.1772465 (PMC13454062; doi:10.3389/fmicb.2026.1772465)
Supplement: Supplementary file 1 [file Table_1.DOCX]

Supplementary Material

# Supplementary Tables S1

| Supplementary table S1. RT-qPCR reaction system and procedure | |
| --- | --- |
| **Reaction System** |  |
| Reagent name | Volumn(ul) |
| 2X One Step RT-PCR Buffer Ⅲ | 12.5 |
| TaKaRa Ex Taq HS (5 U/ μl) | 0.5 |
| PrimeScript RT Enzyme Mix Ⅱ | 0.5 |
| RNase Free dH2O | 5 |
| F(40uM)/R(40uM)/P(10uM) | 0.5/0.5/0.5 |
| Template (RNA) | 5 |
| Total | 25 |

| **Primer and Probe Sequences** | | |
| --- | --- | --- |
| Detection goal | Primer name | Sequence |
|  | GI-F | CGYTGGATGCGNTTYCATGA |
| norovirus-GI | GI-R | CTTAGACGCCATCATCATTYAC |
|  | GI-P | FAM-AGATYGCGATCYCCTGTCCA-BHQ1 |
|  | GII-F | ATGTTCAGRTGGATGAGRTTCTCWGA |
| norovirus-GII | GII-R | TCGACGCCATCTTCATTCACA |
|  | GII-P | FAM-AGCACGTGGGAGGGCGATCG-BHQ1 |

| **Reaction Procedure** |  |  |
| --- | --- | --- |
| Temperature | Time | Cycle |
| 42℃ | 5min | 1x |
| 95℃ | 10s | 1x |
| 95℃ | 5s | 40x, FAM |
| 60℃ | 34s |  |
| 25℃ | 1min | 1x |

**Supplementary Table S2.** Whole-Genome Sequences of representative isolates of 45 common HuNoV genotypes

| Serial number | Genogroup | Genotype | Genotype |
| --- | --- | --- | --- |
| 1 | GI | GI | M87661、OR084255.1、MW243609.1 |
| 2 |  | GI.1[P1] | MT008453.1、MK956173、 |
| 3 |  | GI.2[P2] | MH218656 |
| 4 |  | GI.3[P3] | KY934262.1、MH218647、MT031988.1、PP594195.1 |
| 5 |  | GI.3[P10] | MT008457.1、ON033826.1 |
| 6 |  | GI.3[P13] | MK073892、OR792775.1、MW445537.1、LC769711、MZ021989 |
| 7 |  | GI.3[P14] | MH218730 |
| 8 |  | GI.4[P4] | MN938461.1 |
| 9 |  | GI.5[P4] | OK562729.1、OP669384 |
| 10 |  | GI.5[P12] | MH443711、MT008460.1 |
| 11 |  | GI.6 | MW243609.1、LC726061 |
| 12 |  | GI.7[P7] | KX907730、MT008459.1、LC646335 |
| 13 |  | GI.9[P9] | OP649638 |
| 14 | GII | GII | OR084259.1、OM388564.1 |
| 15 |  | GII.1[P16] | MK753033、MG572182.1、MK483908 |
| 16 |  | GII.1[P33] | OL898506 |
| 17 |  | GII.2 | AF145896、KY421122.1、OK376264.1 |
| 18 |  | GII.2[P2] | MK729086、LC209457、MH671553.1、MT724793 |
| 19 |  | GII.2[P16] | NC_039476.1、KY771081.1、MK614129.1、OK012403、MK614137.1、MN394544.1、OL826943.1、PP267270.1、OP689558、PP267299.1、LC790062 |
| 20 |  | GII.3 | MG601447.1、ON569431、PP645200 |
| 21 |  | GII.3[P12] | KF306213、MT755733、OP901693 |
| 22 |  | GII.3[P16] | KY887598、MG892953、LC769689 |
| 23 |  | GII.3[P21] | OR536499 |
| 24 |  | GII.3[P25] | OL451533.1、OP690505 |
| 25 |  | GII.4 | KX586330.1、MG214988.1、MW981327.1 |
| 26 |  | GII.4 Sydney [P4] | MK073889 |
| 27 |  | GII.4 Sydney[P13] | MW045405 |
| 28 |  | GII.4 Sydney[P16] | MG002631、OQ940068.1、MT029316、MW661278、LC769684、OQ940082.1、OP901694、LC790070 |
| 29 |  | GII.4[P16] | MW305647、OL336389.1、PP267275.1 |
| 30 |  | GII.4[P31] | PP498989.1、LC769701、LC726078 |
| 31 |  | GII.4 Sydney[P31] | MW019959、MT158859.1 |
| 32 |  | GII.4 Hong Kong[P31] | MN400355.2、MT742777 |
| 33 |  | GII.4_San Francisco[P31] | OR262325、OR262328 |
| 34 |  | GII.6[P7] | KU870455.1、OL468714.1、MT158850.1、PP267271.1、LC790056、OQ702254.1 |
| 35 |  | GII.7[P7] | MT731332 |
| 36 |  | GII.8[P8] | MN996298.1、OR546400 |
| 37 |  | GII.9[P7] | MZ312111.1 |
| 38 |  | GII.10[P16] | OP686903 |
| 39 |  | GII.12[P16] | MK355712 |
| 40 |  | GII.13[P16] | LC769703、LC597117.1 |
| 41 |  | GII.13[P21] | MN394545.1 |
| 42 |  | GII.17[P17] | KT970377.1、MN996300.1、MT158861.1、LC644992、PP423020.1、PP267298.1 |
| 43 |  | GII.20[P7] | OQ682536.1 |
| 44 | GIV | GIV.1 | NC_029647、KC894731.1 |
| 45 | GIX | GIX.1[GII.P15] | MT703831.1、MW228040.1、MN462922.1、MT010299.1、OR050586.1 |

**Supplementary Table S4.** Multiplex PCR sequencing reaction system and procedure

**GII group**

**Reaction System(1)**

| Reagent name | Component Name | Volumn(ul) |
| --- | --- | --- |
| Reagent A Mix | 2×Reaction Mix | 25 |
| Reagent B Mix | RT-PCR Enzyme Mix | 2.5 |
| Reagent D1 Mix | Primer 1 | 3 |
| Nuclease-free water |  | 9.5 |
| Template (RNA) |  | 10 |
| **Total** |  | 50 |

**Reaction System(2)**

| Reagent name | Component Name | Volumn(ul) |
| --- | --- | --- |
| Reagent A Mix | 2×Reaction Mix | 25 |
| Reagent B Mix | RT-PCR Enzyme Mix | 2.5 |
| Reagent D2 Mix | Primer 2 | 3 |
| Nuclease-free water |  | 9.5 |
| Template (RNA) |  | 10 |
| **Total** |  | 50 |

**Reaction Procedure**

| Temperature | Time | Cycle |
| --- | --- | --- |
| 50℃ | 30min | 1× |
| 94℃ | 3min | 1× |
| 94℃ | 10s | 35× |
| 68℃ | 6min20s |  |
| 72℃ | 5min | 1× |
| 4℃ | Hold |  |

**Product Purification and Recovery**

The reverse transcription amplification products from Reaction System(1) and Reaction System(2) of the same sample were combined into a single tube, and purification and recovery were performed using 1× AM Pure XP beads.

**GI group**

**Reaction System(1)**

| Reagent name | Component Name | Volumn(ul) |
| --- | --- | --- |
| Reagent A Mix | 2×Reaction Mix | 25 |
| Reagent B Mix | RT-PCR Enzyme Mix | 2.5 |
| Reagent C1 Mix | Primer 1 | 3 |
| Nuclease-free water |  | 9.5 |
| Template (RNA) |  | 10 |
| **Total** |  | 50 |

**Reaction System(2)**

| Reagent name | Component Name | Volumn(ul) |
| --- | --- | --- |
| Reagent A Mix | 2×Reaction Mix | 25 |
| Reagent B Mix | RT-PCR Enzyme Mix | 2.5 |
| Reagent C2 Mix | Primer 2 | 3 |
| Nuclease-free water |  | 9.5 |
| Template (RNA) |  | 10 |
| **Total** |  | 50 |

**Reaction Procedure**

| Temperature | Time | Cycle |
| --- | --- | --- |
| 50℃ | 30min | 1× |
| 94℃ | 3min | 1× |
| 94℃ | 10s | 35× |
| 68℃ | 6min20s |  |
| 72℃ | 5min | 1× |
| 4℃ | Hold |  |

**Product Purification and Recovery**

The reverse transcription amplification products from Reaction System(1) and Reaction System(2) of the same sample were combined into a single tube, and purification and recovery were performed using 1× AM Pure XP beads.

**Supplementary Table S8.** Comparison of performance between MT-capture sequencing and multiplex PCR sequencing in norovirus detection

| Item | MT-capture | | Multiplex PCR |
| --- | --- | --- | --- |
|  | Pre-optimization Process | Optimized Process with the Anti-contamination System |  |
| Probe design scheme | 20–100-nt non-isolength biotinylated oligonucleotides | 20–100-nt non-isolength biotinylated oligonucleotides | 200–300-nt amplicon |
| Duration of the entire process | 8 hours | 7 hours | 8.5 hours |
| Operational convenience | Simple | Simple | Simple |
| Detection sensitivity | High | High | Low |
| simultaneous detection of different genogroups | Y | Y | N |
| Capture efficiency for low viral load samples | Good | Better | Worse |
| Sample multiplexing | Y | Y | N |
| Contamination preventing | N | Y | N |
